# The orexin neuropeptide system: physical activity and hypothalamic function throughout the aging process

**DOI:** 10.3389/fnsys.2014.00211

**Published:** 2014-11-04

**Authors:** Anastasia N. Zink, Claudio Esteban Perez-Leighton, Catherine M. Kotz

**Affiliations:** ^1^Graduate Program in Neuroscience, School of Medicine, University of MinnesotaMinneapolis, MN, USA; ^2^CIMIS, Escuela de Nutricion y Dietetica, Facultad de Medicina, Universidad Andres BelloSantiago, Chile; ^3^GRECC (11G), Minneapolis VA Healthcare SystemMinneapolis, MN, USA; ^4^Department of Food Science and Nutrition, University of MinnesotaSaint Paul, MN, USA

**Keywords:** aging, hypothalamus, energy expenditure, hypocretin, orexin, NEAT, obesity, spontaneous physical activity

## Abstract

There is a rising medical need for novel therapeutic targets of physical activity. Physical activity spans from spontaneous, low intensity movements to voluntary, high-intensity exercise. Regulation of spontaneous and voluntary movement is distributed over many brain areas and neural substrates, but the specific cellular and molecular mechanisms responsible for mediating overall activity levels are not well understood. The hypothalamus plays a central role in the control of physical activity, which is executed through coordination of multiple signaling systems, including the orexin neuropeptides. Orexin producing neurons integrate physiological and metabolic information to coordinate multiple behavioral states and modulate physical activity in response to the environment. This review is organized around three questions: (1) How do orexin peptides modulate physical activity? (2) What are the effects of aging and lifestyle choices on physical activity? (3) What are the effects of aging on hypothalamic function and the orexin peptides? Discussion of these questions will provide a summary of the current state of knowledge regarding hypothalamic orexin regulation of physical activity during aging and provide a platform on which to develop improved clinical outcomes in age-associated obesity and metabolic syndromes.

## Introduction: physical activity and the orexin neuropeptide system

Physical activity can improve overall health. For example, it can prevent obesity and reduce age-associated cognitive decline. There is wide variation between individuals in their drive to be physically active. The drive for physical activity is operationally defined as spontaneous physical activity (SPA). In humans, SPA includes time spent standing and ambulating, but not voluntary exercise. The energy expended by SPA is termed “nonexercise activity thermogenesis” or NEAT. Exercise is a necessary part of a healthy lifestyle but many people cannot or do not exercise. New treatments to target exercise-independent aspects of achieving and maintaining a healthy weight are greatly needed. Spontaneous physical activity is an excellent candidate, but our understanding of the brain mechanisms driving SPA is incomplete. Therapies that enhance SPA will contribute to better clinical outcomes for obesity and metabolic syndrome, diseases of high prevalence in the developed world. This review describes recent advances in our understanding of neuronal processes that regulate SPA, with a specific focus on changes that occur in the orexin neuropeptide system during normal and pathological aging.

**Figure 1:**
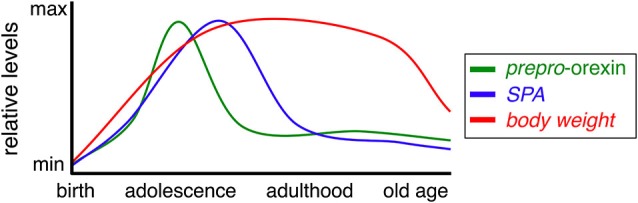
**Prepro-orexin, SPA, and body weight during aging**. Relative levels of prepro-orexin (green), SPA (blue), and body weight (red) throughout the mammalian life span.

The orexin (hypocretin) neurons are a group of hypothalamic neurons defined by expression of the orexin peptides. The orexin signaling system regulates a variety of complex behaviors, including sleep/arousal, reward, food intake and SPA, with an overall effect of increasing energy expenditure. Orexin neuron activity is affected by multiple environmental and physiological variables like fasting and circadian rhythms. Function of the orexin system varies with lifestyle and age (see Figure 1), as does its ability to influence factors that contribute to pathological weight gain in humans and animals. Clarifying how these two variables impact orexin-induced SPA will facilitate development of improved obesity prevention and treatment programs.

### Orexin neuropeptides and receptors

The orexin signaling system consists of two orexin peptides (orexin A and orexin B) and two G-protein coupled receptors (orexin receptor 1, OXR1 and orexin receptor 2, OXR2) (de Lecea et al., 1998; Sakurai et al., 1998). Orexin A and orexin B are 33- and 28-amino acid peptides cleaved from a single gene product, prepro-orexin (Sakurai et al., 1998). Orexin A has equal affinity for both orexin receptors, while orexin B preferentially binds to OXR2 (Sakurai et al., 1998; Ammoun et al., 2003). Both OXR1 and OXR2 couple to the G_q/11_-alpha subunit to activate phospholipase C and induce cation influx, thereby depolarizing neurons and increasing excitability (de Lecea et al., 1998; Zhu et al., 2003). When overexpressed in cultured cells, OXR2 also signals through the pertussis-toxin sensitive G_i/o_-alpha subunit to reduce cAMP production (van den Pol et al., 1998; Zhu et al., 2003). Electrophysiological studies of cell types that endogenously express a single OXR subtype *in vivo* confirm that orexin receptors are generally excitatory in nature and can affect neuronal activity via both presynaptic and post-synaptic mechanisms (Zhu et al., 2003; Aracri et al., 2013; Schöne et al., 2014). Like the other neuropeptide systems lacking known reuptake transporters, it is believed that orexin signaling is terminated through diffusion, receptor sequestration, and enzymatic degradation.

The expression pattern of the orexin receptors differs widely among brain sites but is often complimentary in nature. Most brain sites investigated thus far predominately express a single receptor subtype and those that express both subtypes typically do so in separate cell types (Trivedi et al., 1998; Marcus et al., 2001). Functional differences between the two orexin receptor subtypes are not clearly delineated. Many studies are limited by the use of methods that affect both receptor populations, as is the case with exogenous administration of orexin A and genetic manipulations of the prepro-orexin gene. Direct comparison of OXR1 and OXR2 knockout mice report contributions of both subtypes to body weight and sleep patterns, albeit with one receptor subtype typically displaying a greater effect (Funato et al., 2009; Mieda et al., 2011).

Orexin signaling takes on a modulatory nature in many experimental paradigms. Behavioral or physiological effects differ depending on the brain site of action. In other words, the function of the brain area in which orexin signaling is being manipulated is the primary determinant of the particular orexin-dependent effects that are observed at both the behavioral and cellular levels. For example, orexin A signaling via OXR1 in the periaquaductal gray area induces analgesia through cannabinoid-mediated retrograde inhibition whereas OXR1 signaling in the dorsal hippocampus facilitates excitatory LTP and formation of new associative memories (Ho et al., 2011; Riahi et al., 2013; Yang et al., 2013). Thus, while it is tempting to assign distinct functions to each receptor subtype, the currently available body of data does not fully support a simple, dichotomous characterization. A more refined understanding is needed of functional dissociations in brain-site specific receptor subtypes and the molecular mechanisms underlying them.

### Orexin neurons

In the mammalian brain, orexin neurons are concentrated in the lateral hypothalamus (LH), perifornical area, and dorsomedial hypothalamus (Peyron et al., 1998). Orexin fibers are found throughout the central nervous system (CNS), including nuclei in cortical and limbic areas, basal ganglia, midbrain, brainstem, and spinal cord (de Lecea et al., 1998; Peyron et al., 1998; Taheri et al., 2001; Colas et al., 2014). In addition to orexin, these neurons synthesize glutamate, as well as other neuropeptides, notably dynorphin (Chou et al., 2001; Rosin et al., 2003; Torrealba et al., 2003). Orexin neuron activity is affected by a variety of metabolic signaling molecules (i.e., glucose, leptin, amino acids) and environmental factors which will be discussed in more detail below (Yamanaka et al., 2003; Karnani and Burdakov, 2011; Karnani et al., 2011; Leinninger et al., 2011). For example, activity levels of orexin neurons, as measured by the immediate early gene Fos, increased during the waking phase of the circadian cycle and during fasting or caloric restriction (Sakurai et al., 1998; Estabrooke et al., 2001). Orexin neurons, in turn, regulate physiological and behavioral processes that have major impacts on energy balance and metabolic state, physical activity, blood glucose levels, and food intake (Sakurai et al., 1998; Akiyama et al., 2004; Alam et al., 2005; Kotz et al., 2006; Inutsuka et al., 2014).

As the orexin neurons are known to modulate multiple behaviors, it has been suggested there are functionally specialized subpopulations of orexin neurons, yet this critical issue remains unresolved. The most well-known hypothesis proposes that orexin neurons located in the lateral portion of the LH mediate reward behaviors and those located more medially in the perifornical/dorsomedial areas are involved in arousal and stress (Harris and Aston-Jones, 2006; Harris et al., 2008). This theory is in part supported by the observation that circadian fluctuations in Fos expression in orexin neurons are most pronounced in the medial LH and less so in the more lateral portions, as well as, by differential activation of orexin neurons in reward behavioral paradigms (Estabrooke et al., 2001; Harris and Aston-Jones, 2006; Harris et al., 2008). However, orexin neurons send collateral projections throughout the CNS, indicating that anatomical location of orexin cell bodies is unlikely to be the most informative criterion when attempting to identify or predict functional specialization of orexin neurons. Accordingly, subpopulations of orexin neurons have been described based on electrophysiological and morphological variables (España et al., 2005; Oldfield et al., 2007; Schöne et al., 2011). Analysis of orexin neuron projections to the ventral tegmental area and locus coeruleus revealed that differences in electrophysiological properties and neuronal architecture are better parameters compared to location of soma when attempting to categorize distinct subpopulations of orexin neurons (Schöne et al., 2011; González et al., 2012). While there is some degree of specialization of orexin neurons, the characteristics that define specific subpopulations and whether they have overlapping or unique functions remain poorly defined.

### Orexin and energy expenditure

Orexin peptides modulate energy metabolism, arousal, and physical activity (Chemelli et al., 1999; Hara et al., 2001; Kiyashchenko et al., 2002; Mileykovskiy et al., 2005; Adamantidis et al., 2007; Takahashi et al., 2008; Sasaki et al., 2011; Inutsuka et al., 2014). Orexin system activity is positively associated with activity levels in animals and humans (Kiyashchenko et al., 2002; Wu et al., 2002; Kok et al., 2003). Orexin signaling promotes obesity resistance via enhanced SPA and energy expenditure (Perez-Leighton et al., 2012). Animal models lacking a functional orexin system develop obesity despite consuming fewer calories than their wildtype counterparts (Hara et al., 2001, 2005). Pathological weight gain in these animals is most likely due to energy imbalance resulting from reduced physical activity. Animals in which there is progressive loss of orexin neurons display more severe obesity phenotypes than mice who are only deficient in prepro-orexin, indicating that multiple factors and signaling systems coalesce in orexin neurons to regulate body weight (Hara et al., 2005). To complement genetic ablation approaches, pharmacological studies of repeated orexin A injection into the brain result in body weight loss and protection against obesity (Novak and Levine, 2010; Perez-Leighton et al., 2012; Teske et al., 2013). Indeed, selective activation of orexin neurons in the LH via Designer Receptors Exclusively Activated by Designer Drugs (DREADDs) stimulates SPA, food intake, and energy expenditure (Inutsuka et al., 2014).

Orexin-dependent modulation of SPA involves several brain sites with site-specific participation of OXR subtypes (Kiwaki et al., 2004; Thorpe and Kotz, 2005; Kotz et al., 2006). Data from our laboratory and others show that a major effect of orexin A signaling is to promote SPA and NEAT (Kotz et al., 2006; Inutsuka et al., 2014). Increased SPA and NEAT are observed following injection of the orexin peptides directly into the rostral LH, hypothalamic paraventricular nucleus, nucleus accumbens, locus coeruleus, dorsal raphe nucleus, tuberomammillary nucleus, and substantia nigra (Kotz et al., 2002, 2006; Kiwaki et al., 2004; Thorpe and Kotz, 2005; Novak and Levine, 2010; Perez-Leighton et al., 2012; Teske et al., 2013). Of these sites, our work suggests that orexin A in the rostral LH has the greatest effect on SPA. As this brain area has been the focus of previous reviews the reader is referred to those reviews for additional information (Kotz et al., 2008, 2012; Teske et al., 2010). It is worth emphazing that the effect of orexin A on SPA is a primary outcome that occurs within minutes whereas effects on body weight are considerably more delayed (Teske et al., 2010; Perez-Leighton et al., 2012).

#### Orexin, energy expenditure, and obesity

The strong correlation between orexin signaling, SPA, and NEAT, makes orexin an attractive anti-obesity target. Indeed, selective activation of orexin neurons is sufficient to drive increased SPA and energy expenditure in mice (Inutsuka et al., 2014). Many reports exist implicating reduced physical activity and NEAT in the etiology of obesity in humans (Levine et al., 1999, 2005). Our work using two different animal models of obesity reveals a strong link between endogenous orexin function, SPA, and body weight. In rats selectively bred for their weight gain in response to high-fat diet (HFD), obesity resistant rats have higher sensitivity to the behavioral effects of orexin A (Levin, 1991; Teske et al., 2006, 2013). Over time, HFD decreases SPA in obesity prone animals, whereas obesity resistant rats maintain pre-HFD levels of SPA and sensitivity to orexin-induced SPA (Perez-Leighton et al., 2012, 2013). Additionally, higher SPA in obesity resistant rats predicts lower fat mass gain throughout their lifetime (Teske et al., 2012). Consistent with these findings, non-selectively bred rats that display greater levels of SPA are significantly more resistant to pathological weight gain induced by a HFD compared to animals with naturally lower SPA (Perez-Leighton et al., 2012, 2013). Animals who are resistant to diet induced obesity also exhibit higher expression of prepro-orexin in the LH and enhanced sensitivity to effects of orexin A in rostral LH on SPA (Perez-Leighton et al., 2012, 2013). Importantly, 10 daily treatments of orexin A administration into the rostral LH prevented HFD induced obesity without altering caloric intake (Perez-Leighton et al., 2012). Together, these data implicate orexin signaling in determining sensitivity to diet induced obesity and provide clear evidence that orexins regulate energy expenditure through SPA and NEAT.

Animal models of diet-induced-obesity consistently display attenuated levels of orexin signaling molecules in both the CNS and peripheral tissues (Kotz et al., 2005; Zhang et al., 2005a,b; Sellayah and Sikder, 2014). Similarly, obese humans have lower circulating levels of orexin and impaired orexin receptor activity in adipose tissue (Adam et al., 2002; Digby et al., 2006). No comparable studies have been performed investigating differences in the orexin system in the CNS of obese and healthy humans. Unlike in animal studies, we are unable to distinguish between the contributions of individual differences in orexin signaling that predispose humans to develop obesity, and the consequences of environmental effects of calorie-rich diets and sedentary lifestyles (Kotz et al., 2006; Perez-Leighton et al., 2012, 2013). Nonetheless, physical activity is a promising candidate for improving clinical outcomes in aged humans at both the metabolic and neurological levels (Castaneda et al., 2002; Larson et al., 2006).

#### Orexin, energy expenditure, and narcolepsy

There is a near complete loss of central orexin production in human narcolepsy with cataplexy, as measured by orexin immunoreactivity in post-mortem brain slices (Nishino et al., 2000; Peyron et al., 2000). Human narcoleptic patients suffer from extreme episodes of daytime sleepiness. In both humans and animals, narcolepsy is accompanied by higher BMI, increased prevalence of obesity, and reduced physical activity levels (Daniels, 1934; Hara et al., 2001; Kok et al., 2003; Heier et al., 2011). It should be noted that some research groups have attempted to correlate BMI with orexin levels in blood or CSF, samples which can be relatively easily obtained in a clinical setting. However, studies of circulating orexin, either in serum or CSF, should be interpreted with caution, as one study reported no correlation between orexin A concentrations in serum and CSF samples in either control or narcoleptic patients (Dalal et al., 2001). Here, narcoleptic individuals had normal serum levels of orexin A yet CSF levels were below detectable levels, in agreement with post-mortem tissue analysis showing a widespread loss of orexin production in the hypothalamus (Nishino et al., 2000; Dalal et al., 2001). Perhaps of greater consequence is the issue that measures of freely available orexin neuropeptides do not effectively capture orexin neuropeptide concentrations at important sites of action in the CNS or peripheral tissues nor will this approach fully appreciate the dynamic changes that may be occurring in the signaling system as a whole, including changes in receptor efficacy and cellular excitability (Estabrooke et al., 2001; Kiyashchenko et al., 2002; Wu et al., 2002). Despite these methodological limitations, selective optogenetic or DREADD stimulation of orexin neurons unmistakably rescues deficits in sleep and wake patterns in mouse models of narcolepsy (Adamantidis et al., 2007; Hasegawa et al., 2014).

#### Central orexin and peripheral physiology

As described above, a critically important function of the orexin system is its ability to maintain a healthy energy balance by driving physical activity. Orexins act at sites both in the brain and peripheral tissues to regulate physiological processes that contribute to body weight, notably, glucose mobilization, utilization, and adipocyte differentiation (Cai et al., 1999; Sellayah et al., 2011; Tsuneki et al., 2012). The overwhelming majority of orexin production occurs in the hypothalamus, yet orexin signaling is not limited to the CNS (Sakurai et al., 1998). Small amounts of orexins produced by the enteric nervous system and secretory organs result in circulating plasma levels that are a fraction of those observed in the brain (Sakurai et al., 1998; Kirchgessner and Liu, 1999). Importantly, orexin A given intravenously or intranasally to non-human primates is able to rescue cognitive impairments due to sleep-deprivation, indicating central action of systemically administered neuropeptides and viability of clinical applications (Deadwyler et al., 2007).

Orexin receptors are found in a number of tissues outside of the brain, including adipose tissue, gonads, and the gut (Jöhren et al., 2001; Digby et al., 2006; Ducroc et al., 2007). While most tissues display relatively low levels of orexin receptor expression there is approximately four-fold higher expression of OXR2 in the adrenal glands of rats than of that in the brain (Jöhren et al., 2001). This is consistent with our understanding of the orexin system being involved in HPA-activation and the responses to physiological and environmental stressors. Although the functional significance is unclear, it is worth noting that orexin receptor levels in the adrenal cortex are dysregulated in an animal model of diabetes (Jöhren et al., 2006).

Numerous studies indicate a clear relationship between central orexin signaling and pathological changes in peripheral physiology. Selective loss of orexin neurons in the hypothalamus of mice increases susceptibility to diet-induced obesity and age-related weight gain, despite having an intact orexin system in peripheral tissues (Hara et al., 2001, 2005). As expected, transgenic mice engineered to over-express prepro-orexin, thereby increasing orexin signaling tone, exhibit improved insulin-sensitivity and protection against the negative effects of a HFD on adiposity (Funato et al., 2009). Furthermore, DREADD-dependent activation of orexin neurons in food-deprived mice promoted glucose mobilization into the blood stream, suggesting enhanced ability to access energy stores during a state of energy imbalance (Inutsuka et al., 2014). As a whole, the studies described above demonstrate the importance of orexin signaling in promoting healthy energy balance through coordinated mechanisms in both the CNS and in the periphery.

## Effects of life-style choices on physical activity and the orexin system

Evidence that moderate, aerobic physical activity has positive effects on health and body weight is well established. One of the most well characterized phenomena is the ability of physical activity to improve cognitive performance (Colcombe et al., 2004, 2006; Lindwall et al., 2008; Erickson et al., 2011; Miller et al., 2012). This is a two-way interaction, as choices made throughout life and aging, either directly or indirectly, impact physical activity levels. This section focuses on how excessive calorie consumption (i.e., over-nutrition), which commonly results in obesity and metabolic syndrome, affects physical activity, in particular, SPA, and the orexin system.

In the current climate of rising obesity trends, a great deal of focus has been given to the deleterious effects of sedentary lifestyles on body weight and overall health. Studies have reported that obese individuals spend significantly less time engaged in physical activity. Lean people spend an extra 150 min per day moving compared to obese people, while obese patients sat for 2 h longer per day than lean individuals (Levine et al., 2005). This difference in SPA equates to an additional energy expenditure of 5 kcal/kg in non-obese participants, indicating excellent therapeutic potential for treating pathological body weight (Levine et al., 2005). Severity of obesity (measured as accumulation of fat mass) is negatively correlated with NEAT, although this effect only appears in humans after long-term overfeeding (Levine et al., 1999; Schmidt et al., 2012). These data reinforce the view that obesity decreases physical activity, but there is large inter-individual variability in this effect.

Animal studies support the idea that higher SPA prior to overfeeding, as well as increased SPA during overfeeding, protects against obesity (Teske et al., 2006; Perez-Leighton et al., 2012, 2013). Similarly, development and maintenance of obesity is associated with decreased levels of physical activity in rodents (Bjursell et al., 2008). The question then becomes, what brain mechanisms contribute to obesity via regulation of physical activity levels? Different lines of evidence support the orexin peptides as key modulators of physical activity, especially in response to nutrition levels and energy availability.

The orexin system is well-placed to both modulate and be influenced by metabolic state. Overall, orexin signaling is suppressed in an obese state (Kok et al., 2003; Perez-Leighton et al., 2012). Caloric restriction, as occurs during food deprivation in animals or dieting in humans, increases orexin mRNA and orexin receptor expression (Mondal et al., 1999; Komaki et al., 2001; Alam et al., 2005). Furthermore, orexin neurons act as adaptive glucosensors and are inhibited directly at higher glucose concentrations, suggesting that hyperglycemia results in decreased orexin signaling (Burdakov et al., 2006; Williams et al., 2008; Gonzàlez et al., 2009). This would promote lower SPA and energy expenditure, contributing to the development of obesity, but there are currently no reported electrophysiological studies comparing orexin neuron activity in lean and obese states.

The short- and long-term consequences of diet and lifestyle on orexin neuron activity merit further investigation. It must be emphasized that orexin neurons are part of a local (intra-hypothalamic) and global (across the brain) network involved in the control of behavior and energy balance (Peyron et al., 1998; Burt et al., 2011; Kotz et al., 2012). Thus, when considering specific mechanisms that contribute to obesity, orexin signaling is but one part of an interconnected system influenced by multiple genetic and environmental factors.

## Aging and the orexin system

A number of physiological functions controlled by the hypothalamus vary with age, including SPA, circadian rhythms, and cognitive function. Weight is typically gained throughout early and middle age, followed by gradual, age-associated anorexia (Figure 1, Chumlea et al., 1988; Schoenborn et al., 2002; Sullivan et al., 2002). The evidence reviewed above indicates orexin signaling is an important driver of energy expenditure and modulates energy metabolism via blood glucose levels and food intake. Simply put, increases in physical activity are generally accompanied by greater energy needs. Anecdotally, one might consider the diet of a professional athlete when training compared to off-season calorie consumption. In line with this reasoning, reduced physical activity levels observed in studies of aged humans and animals may underlie decreased appetite and changes in body weight observed in these populations (Meijer et al., 2001; Schoenborn et al., 2002; Kotz et al., 2005; Bordner et al., 2011). Many patients near the end of life undergo precipitous weight loss, suggesting severe dysregulation of mechanisms that normally maintain a healthy body weight (Aziz et al., 2008). Moreover, elderly populations experience a greater prevalence of sleep disturbances and cognitive decline/dementia (Foley et al., 2004; Corrada et al., 2010). The diminished physical activity, blunted circadian rhythms, and cognitive deficits associated with aging could be readily explained by compromised orexin signaling in the aged brain.

### Aging in humans

Reductions in the orexin system are observed in humans under a variety of conditions in which symptom onset and severity are strongly tied to aging (Drouot et al., 2003; Fronczek et al., 2007, 2012; Karakus et al., 2012). Dramatic drops in body weight often precede the rapid cognitive and physical decline seen in age-related neurodegenerative diseases, clearly indicating disruption of neurological and physiological processes that promote healthy energy balance (Fronczek et al., 2007, 2012; Aziz et al., 2008). While it is clear that patients with Parkinson’s and Alzheimer’s disease display significant loss of orexin neurons in post-mortem exams, analysis of CSF levels in living patients do not always bear this pattern, suggesting there may be a progressive and possibly sudden loss of central orexin synthesis or compensatory peripheral production (Ripley et al., 2001; Drouot et al., 2003; Baumann et al., 2005; Fronczek et al., 2007, 2012). Some animal studies suggest a tentative link between neurodegenerative disease symptoms and deficits in orexin signaling in monoaminergic and cholinergic neurons in the brainstem and forebrain (Drouot et al., 2003; Wu et al., 2004; Sakurai et al., 2005; Zhang et al., 2005a,b; Downs et al., 2007; Stanley and Fadel, 2012; Fadel et al., 2013; Yang et al., 2013).

Orexin plasma levels are correlated with body weight in postmenopausal females, such that individuals with more circulating orexin A in their blood have lower BMI (Karakus et al., 2012). However, other studies have failed to identify a clear relationship between changes in orexin CSF and plasma levels. For instance, in narcolepsy, where there is a well-known loss of orexin-producing neurons in the brain, there are reports of patients with low orexin CSF, yet normal orexin plasma levels (Peyron et al., 2000; Dalal et al., 2001). It should be noted that assessments of circulating orexin neuropeptides provide very limited insight into the orexin system as a whole, as they do not accurately reflect the complex minutia of events occurring at vital sites of action in the CNS (see Section Orexin, Energy Expenditure, and Narcolepsy for further discussion). Measuring absolute levels of orexin peptide also fails to capture dynamic changes in orexin receptor signaling or changes in somatodendritic excitability of orexin neurons, which are important factors when considering the overall function of the orexin signaling system. Evidence from non-human primates is in line with this reasoning. There was no detectable difference in orexin B labeling in the LH or serum levels of aged rhesus macaques (25–32 years old) compared to mature adults (9–13 years old), yet there was significantly reduced innervation of orexin B fibers in the locus coeruleus (Downs et al., 2007). Increased levels of orexin in the periphery may be a compensatory response to reduced production in the brain. Therefore, even if peripheral levels of orexin do not decline in aged humans, there may be undetected alterations in prepro-orexin production and/or efficacy of orexin receptor activation in the brain. Unfortunately, given the present lack of investigations using post-mortem human brain tissue or functional imaging, it is still unknown whether age-dependent alterations in physical activity and body composition observed in humans can be attributed to decreased orexin signaling in the CNS.

### Aging in animal models

Animal models exhibit clear age-related reductions in the orexin system in the hypothalamus and other brain regions (Brownell and Conti, 2010; Sawai et al., 2010; Kessler et al., 2011). Aging appears to have a uniform effect on orexin production throughout the hypothalamus as orexin A labeling is reduced to a similar degree in both medial and lateral portions of the hypothalamus (Kessler et al., 2011). Although there is no overt neuronal loss or degeneration in the hypothalamus of aged rats, there is a substantial age-related decrease of both orexin A and orexin B peptides (Sawai et al., 2010; Kessler et al., 2011). Aging also results in reduction of one or both of the orexin receptors in the brain, with some species-specific differences in orexin receptor expression throughout life (Terao et al., 2002; Zhang et al., 2002; Porkka-Heiskanen et al., 2004; Takano et al., 2004). As expected, transgenic mice with enhanced orexinergic tone exhibit resistance to both age-related weight gain and diet-induced obesity (Funato et al., 2009; Willie et al., 2009).

Research groups consistently report reduced behavioral efficacy of orexin-neuropeptides in aged rodents. Intraventricular and intrahypothalamic administration of orexin A increased food consumption in adult rats less than 1 year old, but not in aged, 2-year old rats (Kotz et al., 2005; Akimoto-Takano et al., 2006). The ability of both orexin A and orexin B to alter circadian rhythms and increase time-spent awake was also diminished in aged animals (Morairty et al., 2011). Furthermore, age-related loss of prepro-orexin mRNA production in the LH of rodents is accompanied by reduced orexinergic innervation in the hippocampus, basal forebrain, and locus coeruleus, brain regions associated with cognitive decline in neurodegenerative diseases (Zhang et al., 2005a,b; Downs et al., 2007; Stanley and Fadel, 2012).

Central orexin signaling modulates aspects of peripheral physiology (e.g., blood sugar regulation and adipocity), which are critically linked to obesity and often become dysregulated with age (Cai et al., 1999; Tsuneki et al., 2008, 2012; Sellayah et al., 2011; Inutsuka et al., 2014). Animals that do not produce prepro-orexin in the brain develop insulin sensitivity, hyperglycemia, and increased susceptibility to diet-induced obesity, all of which escalate in severity with age (Cai et al., 1999; Hara et al., 2005; Tsuneki et al., 2008, 2012; Sellayah et al., 2011). Age-associated impairments in brown adipose tissue thermogenesis, which contribute to energy imbalance and weight gain, can be rescued by systemic orexin administration (Sellayah and Sikder, 2014). Aging-dependent reductions in brown adipose tissue thermogenesis are further exacerbated in mice lacking orexin neurons (Sellayah and Sikder, 2014). Importantly, dysregulation of insulin signaling is detected in the hypothalamus of prepro-orexin knockout mice *before* abnormal metabolic symptoms occur in the periphery (Tsuneki et al., 2008). Together, these studies indicate that central orexin neuron dysfunction precedes development of overt changes in peripheral tissues that result in metabolic disorders and pathological weight gain.

The studies described above indicate that orexin release and receptor activation in the brain declines with age, but additional studies are needed to determine if this occurs in a consistent, uniform fashion or if some projections are spared or possibly increased in a compensatory manner (Zhang et al., 2002, 2005a,b; Stanley and Fadel, 2012). This will be an important factor to consider when developing therapies that target orexin signaling, as some treatments may be more or less effective with age.

## Summary

The hypothalamus is an important regulator of energy balance. Orexin neuropeptide-producing neurons in the hypothalamus integrate metabolic cues (energy availability) and physical activity (energy expenditure). Orexin neurons alter their activity in response to metabolic signals from the periphery, including leptin, glucose, and insulin (Håkansson et al., 1999; Moriguchi et al., 1999; Tsuneki et al., 2002, 2012; Yamanaka et al., 2003; Burdakov et al., 2006; Karnani and Burdakov, 2011; Leinninger et al., 2011). Orexin signaling is positively correlated with physical activity and negatively correlated with adiposity in both humans and animals (Hara et al., 2001; Adam et al., 2002; Perez-Leighton et al., 2012). Aging has an overall inhibitory effect on orexin signaling, which is likely exacerbated by unhealthy lifestyle choices (Kok et al., 2003; Hara et al., 2005; Brownell and Conti, 2010; Sawai et al., 2010; Kessler et al., 2011).

While much has been done in animal models and in humans to show that SPA significantly impacts body weight, metabolic and cognitive health, more work is needed to fully understand the neurocircuitry and molecular mechanisms which regulate SPA, in particular, what happens to this network during aging. Given our current knowledge, therapies should be developed that aim at producing behavioral and lifestyle changes that prevent or ameliorate age-associated declines in physical activity. There is a clear need for multifaceted approaches to altering SPA that include targeted manipulations of the neural systems that drive SPA. Knowing that aging is associated with an altered metabolic and hormonal milieu, an important future research direction is to understand how these molecular changes directly impact orexin signaling and SPA.

In summary, hypothalamic orexin activity fluctuates over the lifespan to impact physical activity and body weight throughout the aging process (Figure 1). Aged animals have reduced levels of orexin peptides and receptors, although the magnitude is species dependent. Consistent with a loss of signaling molecules are diminished behavioral, cognitive, and metabolic responses to administration of OXR agonists; a significant issue to consider when developing therapeutics to enhance orexinergic tone. Elevating orexin system activity during aging has the potential to improve both physiologic and cognitive status. A significant strategy in moving forward will be to focus on developing treatments that selectively enhance orexin neuron activity and/or receptor function.

## Conflict of interest statement

The authors declare that the research was conducted in the absence of any commercial or financial relationships that could be construed as a potential conflict of interest.
